# Decreased deposition and increased swelling of cell walls contribute to increased cracking susceptibility of developing sweet cherry fruit

**DOI:** 10.1007/s00425-020-03494-z

**Published:** 2020-11-03

**Authors:** Christine Schumann, Simon Sitzenstock, Lisa Erz, Moritz Knoche

**Affiliations:** Institute for Horticultural Production Systems, Leibniz-University Hannover, Herrenhäuser Straße 2, 30419 Hannover, Germany

**Keywords:** Cell wall swelling, Cellulose, Cracking, Epidermis, Hemicellulose, Pectin, *Prunus avium*

## Abstract

**Main conclusion:**

During fruit development, cell wall deposition rate decreases and cell wall swelling increases. The cell wall swelling pressure is very low relative to the fruit’s highly negative osmotic potential.

**Abstract:**

Rain cracking of sweet cherry fruit is preceded by the swelling of the cell walls. Cell wall swelling decreases both the cell: cell adhesion and the cell wall fracture force. Rain cracking susceptibility increases during fruit development. The objectives were to relate developmental changes in cell wall swelling to compositional changes taking place in the cell wall. During fruit development, total mass of cell wall, of pectins and of hemicelluloses increases, but total mass of cellulose remains constant. The mass of these cell wall fractions increases at a lower rate than the fruit fresh mass—particularly during stage II and early stage III. During stage III, on a whole-fruit basis, the HCl-soluble pectin fraction, followed by the water-soluble pectin fraction, the NaOH-soluble pectin fraction and the oxalate-soluble pectin fraction all increase. At maturity, just the HCl-soluble pectin decreases. Cell wall swelling increases during stages I and II of fruit development, with little change thereafter. This was indexed by light microscopy of skin sections following turgor release, and by determinations of the swelling capacity, water holding capacity and water retention capacity. The increase in cell wall swelling during development was due primarily to increases in NaOH-soluble pectins. The in vitro swelling of cell wall extracts depends on the applied pressure. The swelling pressure of the alcohol-insoluble residue is low throughout development and surprisingly similar across different cell wall fractions. Thus, swelling pressure does not contribute significantly to fruit water potential.

## Introduction

Rain cracking is a critical production problem for many fleshy fruitcrops, especially when rainfall occurs during the later stages of fruit maturation. Sweet cherry and grape are prominent examples of rain-susceptible fruitcrops but many others are also rain-susceptible including: tomatoes, plums, blueberries, currants and gooseberries (Mrozek and Burkhardt 1973; Lichter et al. 2002; Khanal et al. 2011). The economic losses associated with rain cracking in this diversity of fruitcrop species range from a minor impairment of fruit quality due to shallow cracks within the cuticle (microcracks) that can trigger russeting (Knoche et al. 2011) and increase the incidence of fruit rots (Borve et al. 2000) and increase the rate of postharvest water loss (Maguire et al. 1999), to deep cracks (macrocracks) that propagate down through the cell layers of the skin into the flesh opening the way for massive invasion by insects and rots. In this way, macrocracks can destroy an entire crop (Opara et al. 1997; Knoche and Lang 2017).

In sweet cherry, macrocracked fruit are not worth harvesting and so are usually left on the tree where they do not always abscise. These overwintering fruit mummify, and so serve as sources of inoculum for fruit-rot pathogens for the following season’s crop. Rain covers are probably the most effective way to mitigate rain damage in sweet cherries but they do not totally eliminate it and they do involve high levels of capital expenditure.

The appearance of a macrocrack in a sweet cherry is only the final step in a series of events that have already predisposed it to damage from prolonged surface wetness (usually, but not always, associated with rainfall). According to the recent ‘Zipper’ hypothesis (Winkler et al. 2016), these steps include the early cessation of cuticle deposition and the subsequent rapid increase in fruit surface area during final expansion growth (Knoche et al. 2004). The resulting strain in the cuticle can lead to the formation of microscopic cracks (Peschel and Knoche 2005). Exposure to surface wetness and high humidity further exacerbates microcracking (Knoche and Peschel 2006). The microcracks so formed, impair the barrier properties of the cuticle (Borve et al. 2000), allowing highly localized water uptake (Winkler et al. 2016). As a consequence, the cells of the skin and outer flesh that lie immediately beneath a cuticular microcrack, expand rapidly and burst. The giant flesh cells have more negative osmotic potentials and thinner cell walls than the much-smaller, thicker-walled cells of the skin’s epidermis and hypodermis (Grimm and Knoche 2015). Not all cells of the flesh are of the same osmotic potential (Grimm et al. 2020). Hence, those having the most negative osmotic potentials will likely burst first (Grimm et al. 2019). When a cell bursts, it liberates malic acid into the apoplast (Herrmann 2001; Winkler et al. 2015). Here, the malic acid serves to increase the permeability of the membranes of the adjacent cells, and it also weakens their cell walls. This triggers a cascade of cell collapse with further leakage and further damage to adjacent cell walls. This chain reaction we refer to as the Zipper effect.

Malic acid is a common osmolyte in sweet cherries that occurs at a concentration of about 70 mM (Herrmann 2001). Loss of cell turgor causes swelling of both epidermal and hypodermal cell walls (Schumann and Knoche 2020). Together, these cell layers form the structural backbone of the sweet cherry fruit (Brüggenwirth et al. 2014). Cell wall swelling also decreases cell:cell adhesion and so lowers the fracture force of the skin (Brüggenwirth and Knoche 2017). Cuticular microcracks now extend deeper into the skin forming schizogenous macrocracks as epidermal, hypodermal and cortical cells separate one from another along their middle lamellae (Schumann et al. 2019). It would seem that pectins also play a role in these processes as both cell wall swelling and macrocracking are exacerbated by the removal of Ca (e.g. by applications of EGTA; Glenn and Poovaiah 1989) and are inhibited by the application of Ca (e.g. Glenn and Poovaiah 1989).

Occasionally, fruit macrocracking occurs as early as color change (stage II/III) but most macrocracking occurs nearer maturity (late stage III). Increased macrocracking at maturity may result from any of the above processes. The activities of cell wall degrading enzymes also increase at this time (Kondo and Danjo 2001). Little is known about how cell wall swelling changes during fruit development. A better understanding of cell wall swelling requires to quantify swelling in developing fruit and relate changes in swelling to changes in the major cell wall fractions and to the intrinsic swelling behavior of these fractions. Different methods have been used in the past to quantify major cell wall constituents. A well-established procedure is the sequential fractionation of the alcohol-insoluble residue (AIR) of the tissue of interest (Sozzi et al. 2002). The AIR is extracted using a range of solvents (Saulnier and Thibault 1987; Barbier and Thibault 1982; Batisse et al. 1996; Fügel et al. 2004, 2006; Yapo et al. 2007; Yapo and Koffi 2008). The fractions are selectively extracted based on differential solubility of the fraction in the respective solvent. This procedure not only allows to separate pectins, hemicelluloses and cellulose, but also to fractionate pectins into water-soluble pectins (WSP), oxalate-soluble pectins (OXP), HCl-soluble pectins (HSP) and an NaOH-soluble pectins (OHP). Chemical analyses of the extracted fractions confirmed the identity of the fractions in a range of plant species including grapes (Saulnier and Thibault 1987), sour cherries, strawberries and apples (Fügel et al. 2004), sweet cherry (Batisse et al. 1996), sugar beet (Rombouts and Thibault 1986), passion fruit (Yapo and Koffi 2008) and citrus (Yapo et al. 2007). The objectives of this study are: (1) to identify any changes in the major cell wall fractions in developing sweet cherry fruit, (2) to quantify cell wall swelling during fruit development and (3) to identify which cell wall fractions account for cell wall swelling during fruit development. We focus on sweet cherry as a model for fleshy fruit because of the large body published information already available on this species.

## Materials and methods

### Plant material

Developing sweet cherry fruit (*Prunus avium* L.) of the cultivar ‘Regina’ were sampled weekly from 33 to 96 days after full bloom (DAFB). The cultivars ‘Adriana’, ‘Burlat’, ‘Dönissens Gelbe’, ‘Earlise’, ‘Fabiola’, ‘Hedelfinger’, ‘Kordia’, ‘Merchant’, ‘Regina’, ‘Sam’, ‘Samba’, ‘Schneiders Späte’, ‘Staccato’, and ‘Sweetheart’ were sampled at the beginning of pit hardening (stage II) and at maturity (stage III). The timings for each cultivar were judged based on fruit size and color. All trees were cultivated under a rain shelter at the Horticultural Research Station of the Leibniz University in Ruthe (lat. 52°14′ N, long. 9°49′ E). Trees were grafted on ‘Gisela 5′ rootstocks (*Prunus cerasus* × *P. canescens*). Unless otherwise stated, fruit were processed fresh on the day of sampling or stored at − 20 °C pending extraction of cell walls. Fruits used in the experiments were selected for uniformity of development based on size and color and freedom from visual defects.

Fruit and pit fresh and dry weights were recorded. For dry weight, fruit and pits were dried at 103 °C to constant weight and the dry weight taken. Further, the osmolarity of juice extracted using a garlic press was quantified by water vapor pressure osmometry (VAPRO^®^ 5600, Wescor, Logan, UT). All determinations were carried out with ten replicates except for the fruit fresh weight, where 50 replicates were used.

### Light microscopy

Cell wall swelling was determined using the procedure described in detail by Schumann and Knoche (2020). Briefly, epidermal skin sections (ES) were prepared from a fruit’s equator in the cheek region. Skin strips were excised (3 mm wide) using parallel-mounted razor blades and the ES cut as thin sections parallel to the surface. The ES were blotted using soft tissue paper, positioned on a microscope slide in a drop of deionized water, transferred to the stage of a microscope (BX-60, Olympus, Hamburg, Germany) and inspected at 40×. Calibrated digital photographs (camera: DP73; Olympus) were taken and the thicknesses of anticlinal cell walls quantified by image analysis (cellSens; Olympus Soft Imaging Solutions; Münster; Germany). The measurement was of the cell wall thickness between two turgid living cells. It thus comprised the sum of the walls of two neighboring cells plus the intervening pectin middle lamella. Earlier studies (Schumann and Knoche 2020) had established that cell wall swelling does not occur in turgid cells—it would seem cell turgor somehow prevents wall swelling. Therefore, to quantify swelling in vivo, cell turgor in the ES was released by exposing it to a freeze/thaw cycle. Following equilibration at room temperature, cell wall thickness was again measured between two now-flaccid cells. Swelling was quantified as the difference between the thickness measured before turgor release from that measured just after turgor release. One ES was cut from each of ten replicate fruit per treatment, two micrographs were taken per ES and two cell walls were measured per micrograph. The number of observations was thus 40 per treatment (10 × 2 × 2).

### Cell wall extraction and fractionation

Cell walls were extracted as the AIR using the protocol of Sozzi et al. (2002) with minor modifications. Briefly, ten replicate fruit were pitted and the remaining skins and flesh were homogenized for 2 min in 4 ml of ice-cold ethanol (80%, v:v) per g of tissue. The homogenate was then boiled for 30 min, cooled and filtered through glass filter paper (Whatman GF/C). The insoluble residue was then washed with 95% (v:v) ethanol and re-filtered. Next, the residue was extracted for 15 min with 3 ml of chloroform:methanol (1:1, v:v) per g tissue, filtered and re-washed with the same solvent mixture, followed by a final wash with acetone. The resulting AIR was dried overnight, weighed and stored over dry silica gel. The developmental time course was established with six biological replicates for ‘Regina’. All other comparisons were carried out with three replications.

The AIR of developing ‘Regina’ fruit was fractionated using a standard protocol (e.g. Rombouts and Thibault 1986; Saulnier and Thibault 1987; Barbier and Thibault 1982; Batisse et al. 1996; Fügel et al. 2004, 2006; Yapo et al. 2007; Yapo and Koffi 2008). The total AIR of ten fruit was suspended and stirred for 30 min at 40 °C in 50 ml deionized water per 0.8 g of AIR. The slurry was centrifuged (Sorvall RC-5B Plus; Thermo Scientific, Waltham, Massachusetts, USA) at 14,000 g for 25 min at 20 °C. The supernatant was removed and kept separate. The procedure was repeated, except that the pellet was now re-suspended and stirred in deionized water for 1 h at 40 °C. The aqueous supernatants were then combined, dialyzed (Carl Roth, Karlsruhe, Germany, MWCO: 14,000) for 2 d against deionized water at room temperature and lyophilized for 6 d. The fraction obtained represents the water-soluble pectins (WSP). Next, a series of four extractions was carried out to create a series of differentially soluble cell wall fractions. After each extraction, the slurry was centrifuged, the supernatant retained and the pellet re-suspended in the next solvent. The sequence of the extraction series was (1) in 50 ml 0.5% (w:v) NH_4_-oxalate solution per 0.8 g AIR for 90 min at 40 °C (the oxalate-soluble pectins, OXP), next (2) in 0.05 M HCl for 90 min at 60 °C (the HCl-soluble pectins, HSP), next (3) in 0.05 M NaOH for 90 min at 30 °C (the NaOH-soluble pectins, OHP) and last (4) in 16% (w:w) aqueous NaOH for 90 min at 30 °C (the hemicelluloses, HC). The pellet remaining after the final extraction represents the cellulose fraction (CL) plus some minor amounts of lignin originating from the fruit’s xylem.

The pellets were then washed twice with 100 ml deionized water, resuspended and re-centrifuged, the appropriate supernatants were combined and lyophilized as described above. The supernatants of both NaOH extraction steps were adjusted to pH 6.5 with HCl prior to dialysis. The pellet of the remaining CL fraction was suspended in 50 ml distilled water, dialyzed and lyophilized. The dry weight of each fraction was recorded after lyophilization and the fractions stored above dry silica gel. The whole procedure was done in triplicate.

On a single occasion a cell wall extraction was carried out of an exocarp-enriched tissue sample vs. a mesocarp tissue sample. The purpose of this separation was to identify potential spatial heterogeneity in the swelling of cell walls extracted from the skin (exocarp) and from the flesh (mesocarp). For this, the fruit was taken from the same lot of mature ‘Regina’ fruit. These were peeled and pitted while still frozen. Because it is technically impossible to separate exocarp and mesocarp in sweet cherry, the peel represents the exocarp-enriched tissue sample (with some adhering mesocarp), the remaining flesh the pure mesocarp tissue sample (no adhering skin) (Alkio et al. 2012). For comparison 3 × 20 fruit were pitted and the exocarps and mesocarps processed together. The frozen tissue samples were lyophilized and ground with pestle and mortar. The extractions were carried out as described above with three replications of 20 fruit each.

### Hydration properties

The hydration properties as indexed by the swelling capacity, the water holding capacity and the water retention capacity (Raghavendra et al. 2004; Basanta et al. 2013) were estimated for the AIR of developing ‘Regina’ fruit. Values for the swelling capacity and the water retention capacity were also determined for the five cell wall fractions OXP, HSP, OHP, HC and CL, each at six stages of fruit development. The hydration properties were estimated according to procedures described previously, with minor adjustments (Raghavendra et al. 2004; Basanta et al. 2013). All determinations were carried out using three replicates. Briefly, for the determination of the swelling capacity, 50 mg (± 0.1 mg) of the AIR or 25 mg of the respective cell wall fraction were weighed in a graduated conical glass tube and 12.5 ml of deionized water was added. To remove any entrapped air and to ensure thorough wetting of the samples, the tubes were vacuum infiltrated (3 kPa) three times, for 10 min each. To improve reproducibility of the determinations, each tube was stirred once, 6 h after the start of incubation. After a total equilibration time of 20 h at room temperature, the final swollen volume of the AIR and of the respective fractions was recorded. The swelling capacity (SC) was calculated as:

$${\text{SC }}\,\left( {{\text{ml}} \times {\text{g}}^{ - 1} } \right) = \frac{{{\text{Volume of swollen AIR}} \left( {{\text{ml}}} \right) }}{{{\text{Original sample dry weight}} \left( {\text{g}} \right)}}.$$

For the determination of the water holding capacity, 50 mg (± 0.1 mg) of the AIR was weighed into a glass tube and 12.5 ml of deionized water added as described above. Following vacuum infiltration and equilibration for 20 h, the supernatant was removed and the weight of the wet pellet recorded. The wet pellet was dried to constant weight at 70 °C and the dry weight recorded. The water holding capacity (WHC) was calculated as:

$${\text{WHC}} \left( {{\text{g}} \times {\text{g}}^{ - 1} } \right) = \frac{{{\text{Weight of wet pellet}} \left( {\text{g}} \right) - {\text{Weight of dry pellet}}\left( {\text{g}} \right)}}{{{\text{Weight of dry pellet}}\left( {\text{g}} \right)}}.$$

For the determination of the water retention capacity, we used the same procedure as for the water holding capacity, except for an additional centrifugation (30 min at 2000*g*) before removal of the supernatant. Thus, the water holding capacity also includes loosely associated water, whereas the water retention capacity is an index for more strongly bound water (Basanta et al. 2013).

For the water retention capacity, the mass of the cell wall fraction was reduced to 25 mg instead of 50 mg for the AIR sample. The cell wall fractions were also lyophilized for 3 d instead of oven drying. The water retention capacity (WRC) was calculated as:

$${\text{WRC}} \left( {{\text{g}} \times {\text{g}}^{ - 1} } \right) = \frac{{{\text{Weight of wet centrifuged pellet}} \left( {\text{g}} \right) - {\text{weight of dry pellet}} \left( {\text{g}} \right)}}{{{\text{Weight of dry pellet}} \left( {\text{g}} \right)}}.$$

### Swelling pressure

The swelling pressure was determined for the AIR samples for the developing sweet cherry fruit and also for the OXP, HSP, OHP, HC and CL fractions of mature fruit. The procedure described previously was used (Schumann and Knoche 2020). Briefly, AIR (25 mg per replicate for the developmental time course and the exocarp/mesocarp comparison, 20 mg per replicate for comparing fractions at maturity) was placed on a stainless steel frit in a custom-built pressure chamber (inner diameter 25.5 mm). The cell wall was wetted using 70% (v/v) aqueous ethanol supported by vacuum infiltration (20 kPa for 10 min). Initial experiments had established that a concentration of 70% ethanol substantially prevented swelling. Using a universal material testing machine (BXC-FR2.5TN; Zwick GmbH & Co. KG, Ulm, Germany; 50 N force transducer, KAP-Z; Zwick/Roell), a plunger was placed on the hydrated cell wall in the pressure chamber. The plunger was fitted with a 25 mm diameter stainless steel frit. Using this setup, the cell wall sample was pressurized at 5.2 N. When this pressure was reached, the 70% ethanol was replaced by deionized water to initiate swelling. At this point, the minimum height of the cell wall sample was recorded to calculate its minimum volume (${V}_{\mathrm{min}}$). The pressure was held at 5.2 N for 12 h. Subsequently, the pressure was reduced stepwise to 2.5, 1.0, 0.5, 0.25, to 0.1 N, each pressure step followed by a 12 h holding period. As pressure was decreased, cell wall volume increased (swelled). The extent of cell wall swelling was recorded by the material testing machine’s displacement transducer and the applied pressure was recorded by its force transducer. From the position change of the plunger, the volume change ($\Delta V$) due to swelling was calculated. The swelling pressure (${P}_{0}$) of the cell wall sample was calculated as the intercept of a plot of the maximum change in volume ($\Delta V$) at any one pressure vs. the natural logarithm of the applied pressure. Swelling pressures were determined using three replicate machine runs, where each run involved an independent extraction or fractionation sample.

### Data analyses and statistics

Data are presented as means ± standard errors. Where not shown, error bars are smaller than the data symbols. Data were analyzed statistically by analysis of variance (Proc GLM) or by regression analysis (Proc REG) using the statistical software package SAS (version 9.1.4; SAS Institute, Cary, NC). Mean comparisons were made using the Tukey Studentized Range Test at *P* < 0.05 using R (packet multcomp 1.4–0, procedure glht, R3.0.2; R Foundation for Statistical Computing, Vienna, Austria). Pearson correlation coefficients were calculated using R.

## Results

### Fruit growth and the deposition of cell wall

**Fig. 1:**
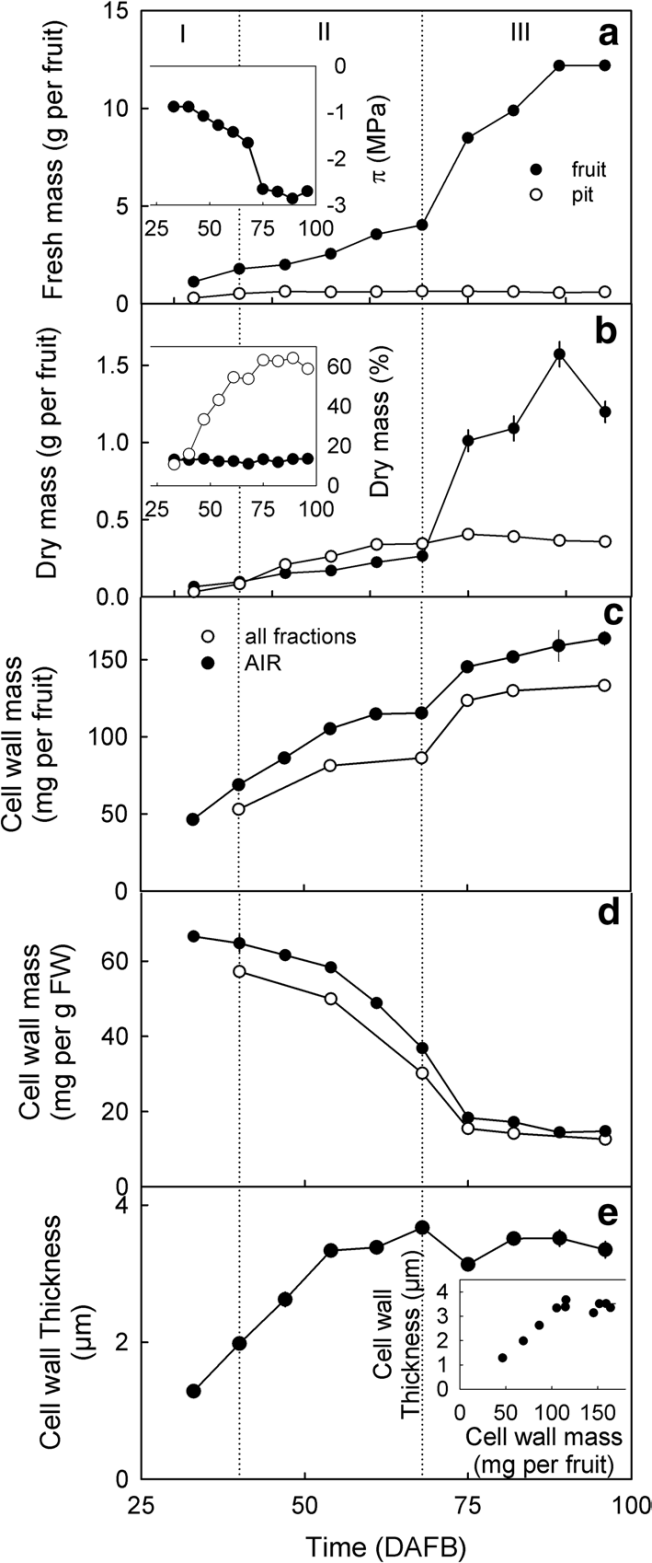
Developmental time course of fruit growth (**a**, **b**), osmotic potential (Inset in **a**), dry matter content (Inset in **b**) deposition of cell wall material determined as the alcohol-insoluble residue (‘AIR’) and the total cell wall mass calculated as the sum of the extracted cellulose, hemicellulose and all pectin fractions on a per fruit basis (**c**) and on a unit fresh mass basis (**d**). **e** Developmental time course of cell wall thickness of epidermal cell walls as determined by light microscopy. Inset in **e**: Relationship between the mass of AIR on a per fruit basis and the cell wall thickness. Fruit growth was indexed as the increase in fresh mass (**a**) and dry mass (**b**) of fruit and pit. Time scale in days after full bloom (DAFB)

**Fig. 2:**
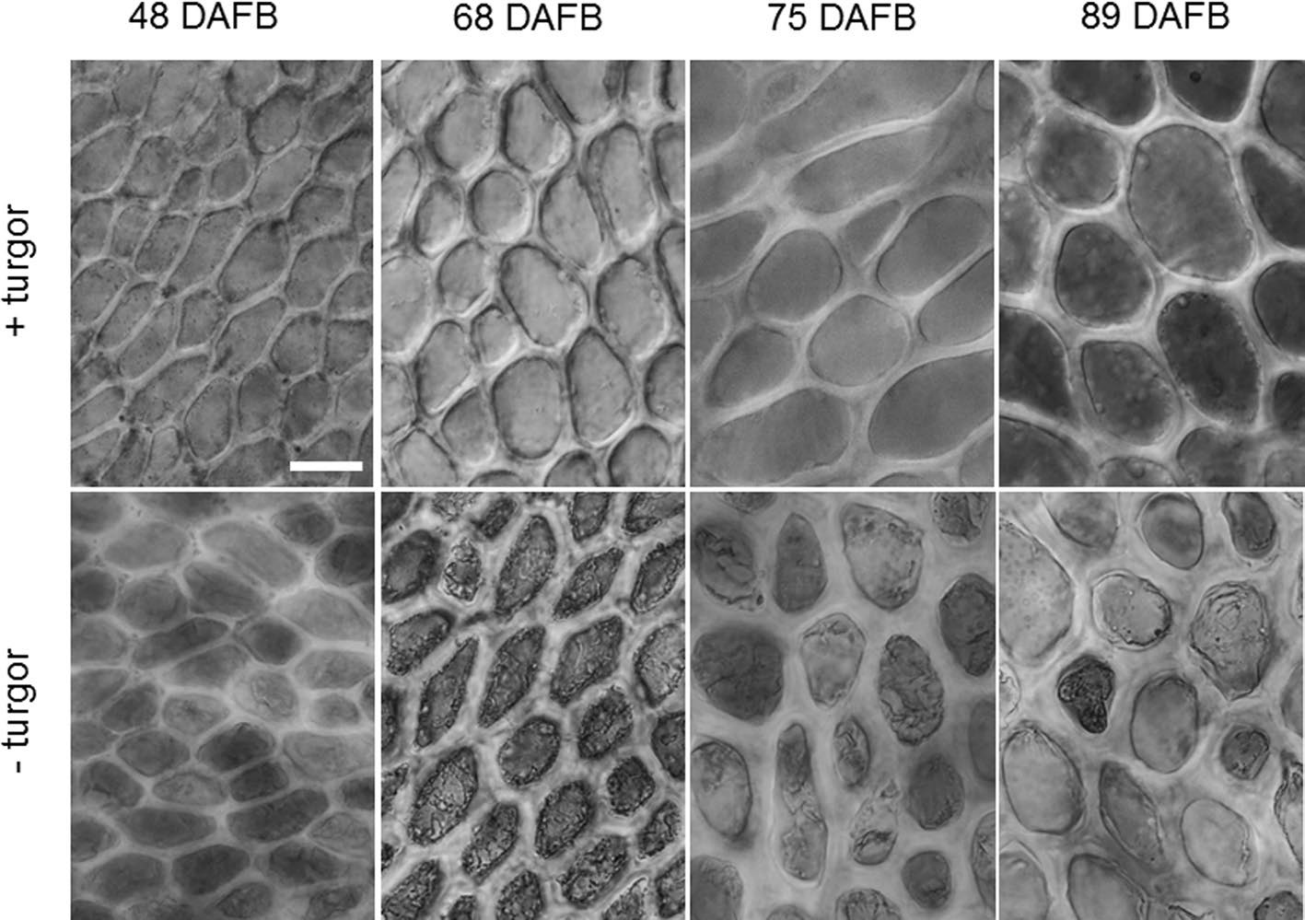
Micrographs of the time course of change in thickness of anticlinal cell walls of excised epidermal skin sections before (top row) and after release of turgor (bottom row) 48, 68, 75 and 89 days after full bloom (DAFB). Bar 20 µm

Fruit fresh and dry masses typically increased in a double-sigmoidal pattern with time characterized by stages I, II and III of fruit development (Fig. 1a, b). Pit masses increased until about mid stage II and then remained constant. The dry mass content of the flesh was also constant throughout development, whereas the dry mass of the pit increased. The osmotic potential decreased (became more negative), particularly during stage III (Fig. 1a (inset)). During fruit development, and on a whole-fruit basis, the total mass of cell wall material increased (Fig. 1c). The increase in cell wall mass failed to keep pace with the increase in fresh mass as indexed by a marked decrease, particularly during stage II and early stage III (Fig. 1d). Inspection of the light microscope images revealed that the thickness of cell walls of epidermal cells increased during stages I and II but then remained constant until maturity (Figs. 1e and 2).

The exocarp accounted for 27% of the total cell wall mass of a fruit, and the mesocarp for the remaining 73% (Table 1). The cell wall mass, on a per-fresh-weight basis, was 2.7-fold higher in the exocarp than in the mesocarp. Despite the large differences in cell wall mass, exocarp and mesocarp had very similar swelling properties (swelling capacity, water holding capacity, water retention capacity and swelling pressure). These did not differ significantly from those of the mean AIR sample obtained for whole (pitted) fruits.

**Table 1:** Tissue fresh mass and cell wall mass after extraction of alcohol-insoluble solids (AIR) of different tissues at 96 days after full bloom and the hydration properties water holding capacity (WHC), swelling capacity (SC), water retention capacity (WRC) and swelling pressure

Tissue	FW_tissue_ (g per fruit)	Cell wall mass		WHC (g g^−1^)	SC (ml g^−1^)	WRC (g g^−1^)	Swelling pressure (kPa)
		(mg per fruit)	(mg per g FW_tissue_)				
Exocarp	1.0 ± 0 a^a^	39.0 ± 1 a	38.6 ± 1 a	37 ± 1 a	33 ± 2 a	24 ± 2 a	12 ± 1 a
Mesocarp	7.6 ± 0 b	107.3 ± 4 b	14.2 ± 1 b	41 ± 5 a	38 ± 2 a	21 ± 1 a	10 ± 0 a
Whole fruit^b^	10.6 ± 0 c	164.2 ± 2 c	15.4 ± 0 b	39 ± 2 a	33 ± 1 a	19 ± 0 a	11 ± 2 a

Data present means ± SE. Swelling properties were determined on 25 mg of the AIR of the respective tissue

^a^Mean separation within columns by Tukey’s Studentized range test, *P* < 0.05

^b^Pitted fruit. Different fruit from same batch

**Fig. 3:**
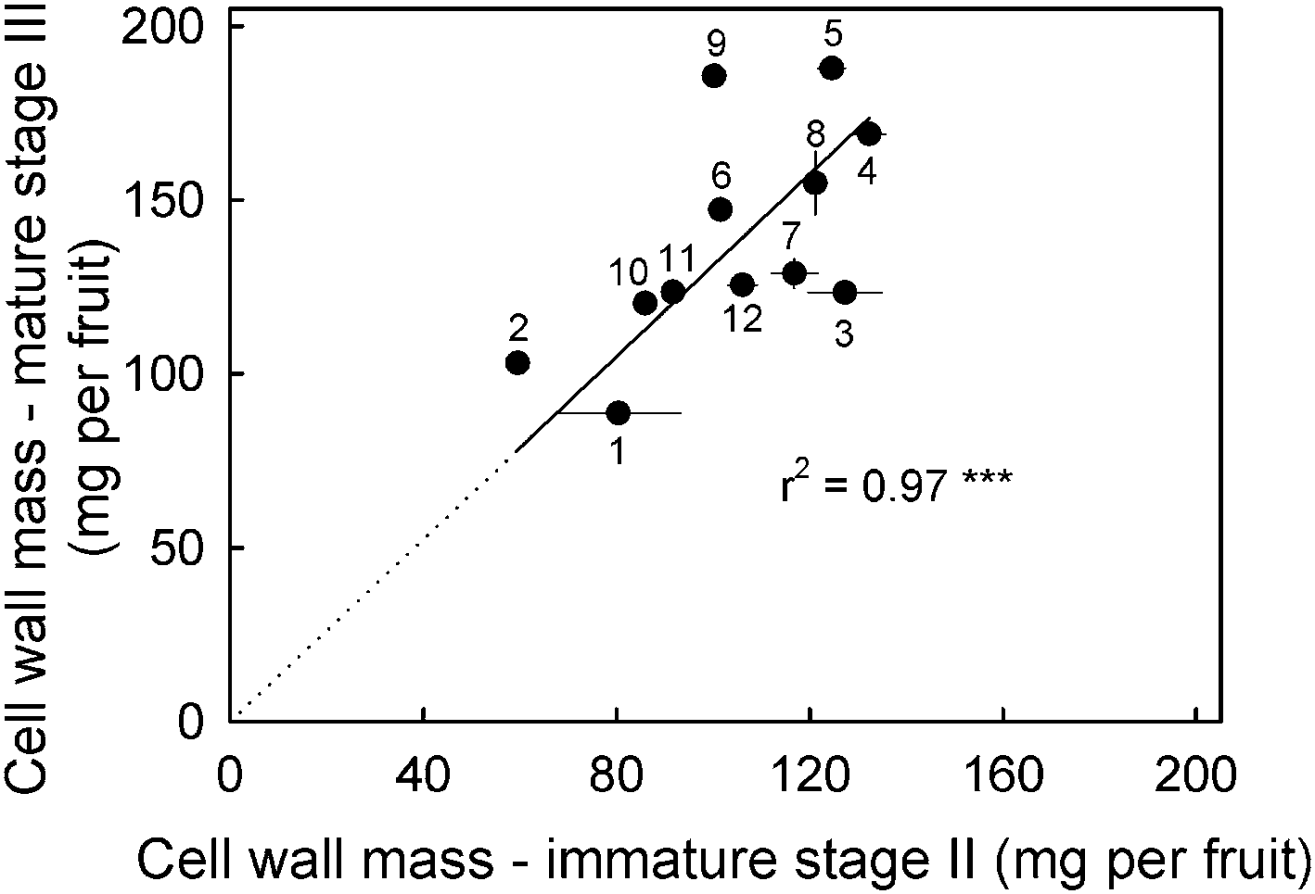
Relationship between the amount of cell wall mass per fruit at the fully mature (stage III) and the immature stage II. Data symbols represent means of different sweet cherry cultivars. The cultivars were Adriana (1), Dönissens Gelbe (2), Early Korvic (3), Fabiola (4), Gill Peck (5), Hedelfinger (6), Kordia (7), Rainier (8), Regina (9), Sam (10), Schneiders (11) and Sweetheart (12). The y-axis intercept of the regression line was not significantly different from zero. Hence, the regression line was forced through the origin. The equation was: Cell wall mass ripe (mg per fruit) = 1.31 ± 0.07 × Cell wall mass unripe (mg per fruit), *r*^2^ = 0.97***

The cell wall masses of immature vs. mature fruit were significantly related across the 12 different sweet cherry cultivars (Fig. 3). The slope of the regression line was 1.31 indicating that (averaged across cultivars) the cell wall mass had increased by 31% (1.31-fold) from stage II to mature stage III. In the same interval fruit fresh mass had increased about 300% (about threefold) from 3.4 ± 0.3 g to 10.0 ± 0.5 g per fruit).

### Developmental time course of major cell wall constituents

**Fig. 4:**
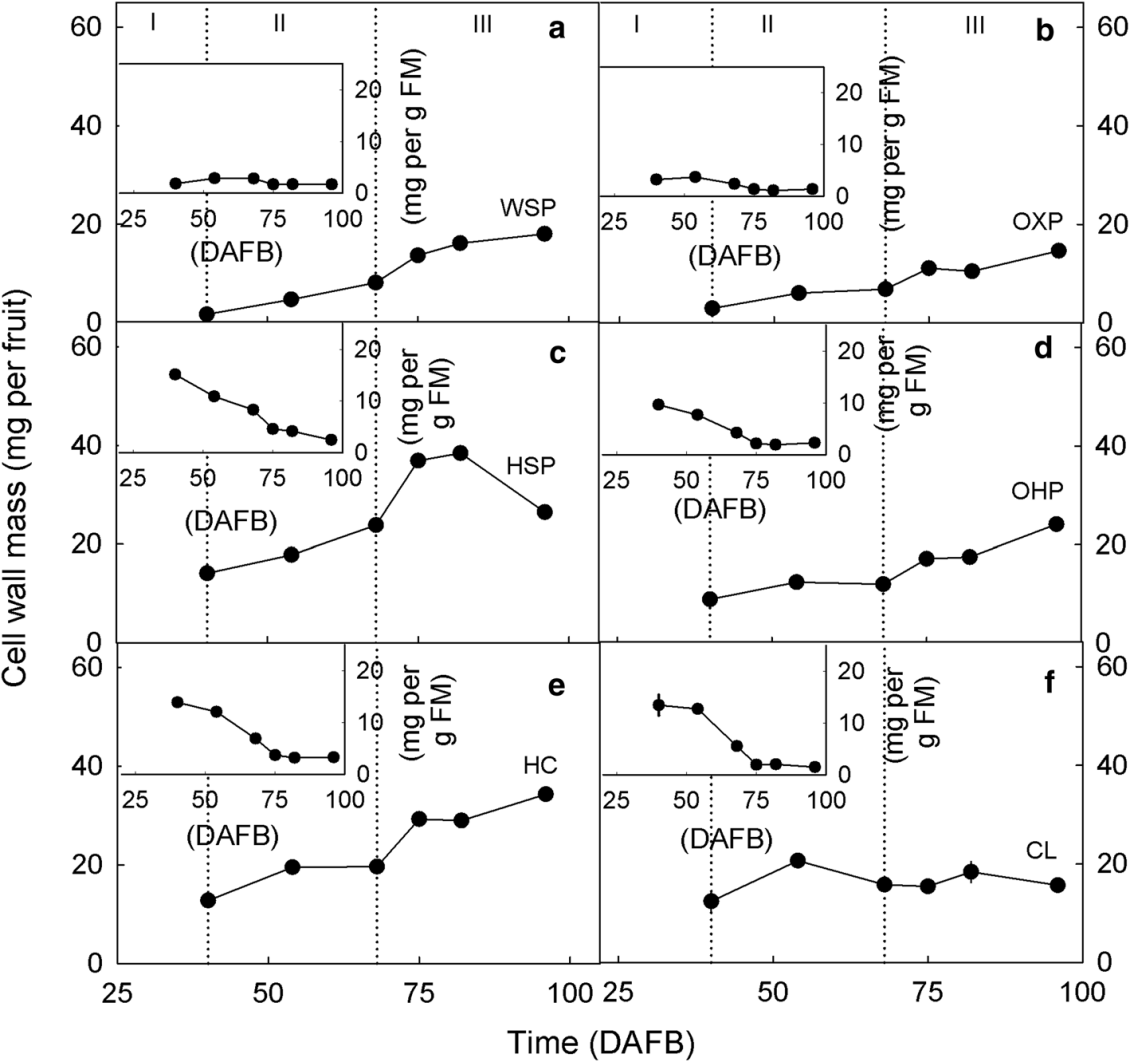
Developmental time course of the deposition of various pectin fractions on a per fruit basis (main graphs) and on a unit fresh mass basis (insets). **a** Water-soluble pectins (WSP). **b** Oxalate-soluble pectins (OXP). **c** HCl-soluble pectins (HSP). **d** NaOH-soluble pectins (OHP). **e** Hemicelluloses (HC). **f** Cellulose (CL). Time scale in days after full bloom (DAFB)

**Fig. 5:**
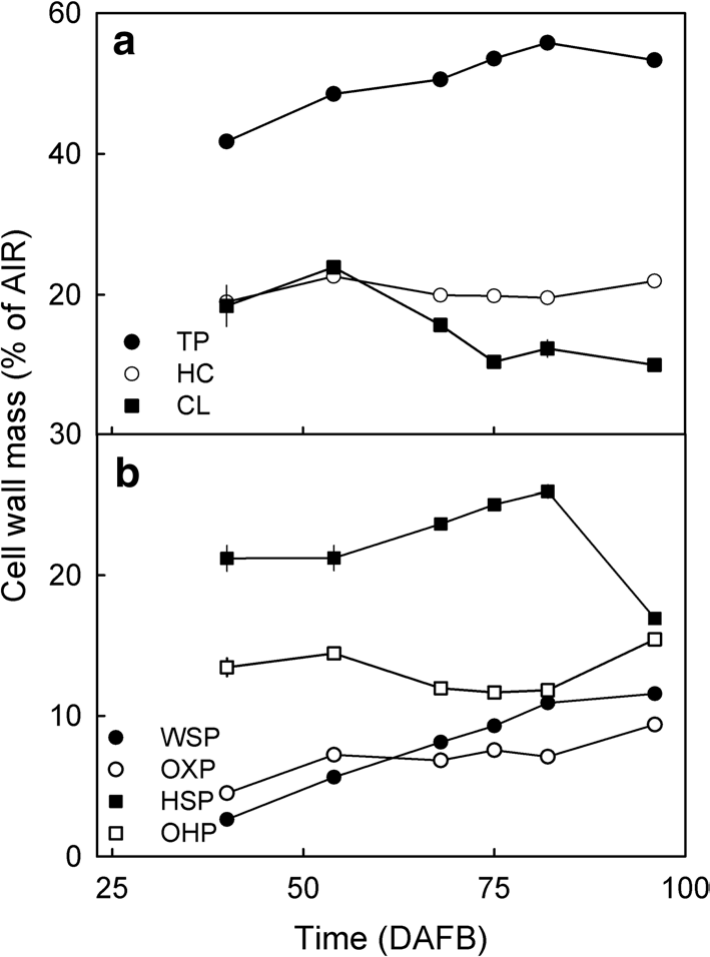
Developmental time course of the change in composition of the cell wall material determined as the alcohol-insoluble residue (‘AIR’). **a** Total pectins (‘TP’), hemicelluloses (‘HC’) and cellulose (‘CL’). **b** Water-soluble pectins (‘WSP’), oxalate-soluble pectins (‘OXP’), HCl-soluble pectins (‘HSP’) and NaOH-soluble pectins (‘OHP’). The TP was calculated as the sum of the WSP, OXP, HSP and OHP

Marked changes occurred in the cell wall fractions of developing fruit (Fig. 4). Here, the WSP, OXP, OHP, and HC all increased during stages II and III (Fig. 4a, b, d, e, f). The HSP increased only until about 82 DAFB and then decreased, the CL remained about constant (Fig. 4c, f). Pectins represented the largest fraction within the AIR. Their contribution to the AIR increased, whereas that of the HC remained constant and that of the CL decreased (Fig. 5a). At maturity the individual fractions averaged 11.6% (WSP), 9.4% (OXP), 16.9% (HSP), 15.4% (OHP), 21.1% (HC) and 9.7% (CL) of the total AIR (Fig. 5). Expressed as fractions of the total pectins, these values were 21.7% (WSP), 17.6% (OXP), 31.7% (HSP) and 29.0% (OHP).

On a per-gram-fresh-mass basis, all pectin fractions (except for WSP) and the HC and CL decreased during fruit development, indicating that the increasing fruit fresh mass ‘diluted’ the pectin fractions (Fig. 4, insets).

It is worth noting that averaged across development, the sum of all fractions amounted to about 85% of the AIR, indicating that only minor losses occurred during fractionation (Fig. 1c).

### Swelling of cell walls and its major constituents

**Fig. 6:**
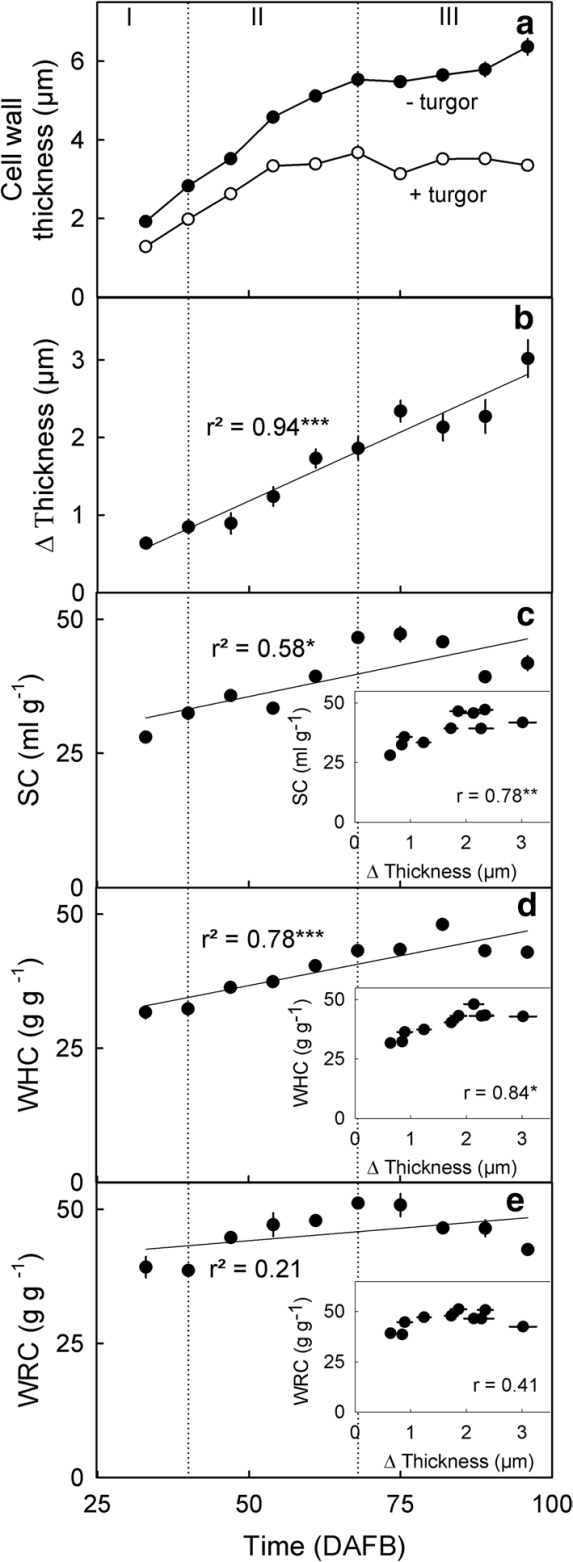
**a** Developmental time course of the change in thickness of epidermal cell walls before (‘+ turgor’) and after removal of turgor (‘− turgor’). Swelling was induced by releasing turgor using a freeze/thaw cycle. Inset: Cell wall swelling (‘Δ Thickness’) calculated as the difference between the cell wall thickness before and after release of turgor. **b**, **c**, **d** Developmental time courses of change of hydration properties of cell wall extracts as indexed by the swelling capacity (SC) (**b**), the water holding capacity (WHC) (**c**), and the water retention capacity (WRC). Cell wall extracts were prepared by extraction with ethanol (alcohol-insoluble residue, AIR). Insets in **b, c, d**: relationship between the SC, WHC and WRC, respectively, and cell wall swelling. Hydration properties and cell wall swelling were determined on fruit from the same batch. Time scale in days after full bloom (DAFB). For calculation of SC, WHC and WRC see Materials and Methods

Following the release of turgor by a freeze/thaw cycle, cell wall thickness increased 1.3–1.9-fold depending on stage of fruit development (Fig. 6a). Calculating the extent of cell wall swelling revealed a near-linear increase in thickness during fruit development (Fig. 6b). Similarly, the swelling capacity, the water holding capacity and the water retention capacity of cell wall extracts increased up to the stage II/III transition, and then remained constant (swelling capacity and water holding capacity, Fig. 6c, d) or decreased (water retention capacity, Fig. 6e). Values of the swelling capacity and of the water holding capacity were significantly correlated to cell wall swelling (Fig. 6c, d, insets). Only the relationship between the water retention capacity and cell wall swelling was not significant (Fig. 6e, inset). Furthermore, the water holding capacity (*r* = 0.91***) and the water retention capacity (*r* = 0.76*) were significantly correlated with the swelling capacity.

**Fig. 7:**
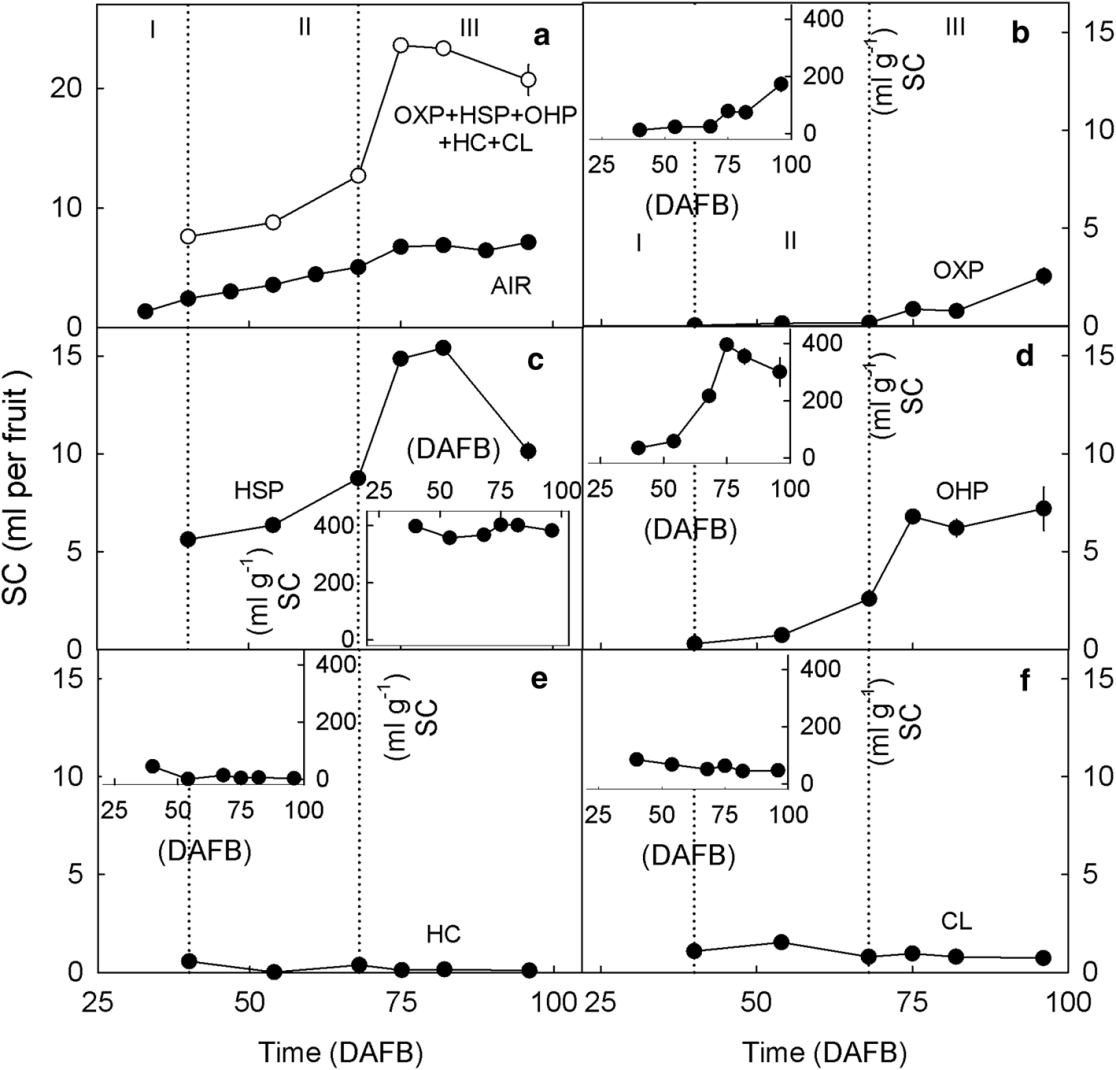
Developmental time course of change in the swelling capacity (SC) of different cell wall fractions extracted from developing sweet cherry fruit. The SC was calculated on a whole fruit basis (Main graphs **a**–**f**) and on a unit dry mass basis of the respective cell wall fraction (Insets **b**–**f**). **a** Alcohol-insoluble cell wall residue (AIR) and the sum of oxalate-soluble pectins (OXP), HCl-soluble pectins (HSP), NaOH-soluble pectins (OHP), hemicelluloses (HC) and cellulose (CL). **b** OXP. **c** HSP. **d** OHP. **e** HC. **f** CL

On a whole-fruit basis the swelling capacity of the AIR increased during each developmental stage. Calculated as the sum of the intrinsic swelling capacity of each single fraction (except for WSP), the swelling capacity increased until about 75 DAFB and then decreased slightly (Fig. 7a). In the in vitro assays, the WSP will be in solution in the supernatant and, therefore, its swelling capacity (and the water retention capacity) cannot be determined in vitro*.* The sum of the swelling capacities of the remaining fractions was on average 3.0-times higher than the swelling capacity of the AIR.

The fraction that had the highest intrinsic swelling capacity was the HSP followed by the OHP and then the OXP (Fig. 7b, c, d). Two factors account for this: (1) the large swelling of the HSP and OHP fractions (Fig. 7c, d; insets) and (2) their large mass fraction on a per-fruit basis, in case of the HSP. The intrinsic swelling capacities of the HC (Fig. 7e) and the CL (Fig. 7f) were low, both when expressed on a unit-mass basis and on a per-fruit basis.

**Fig. 8:**
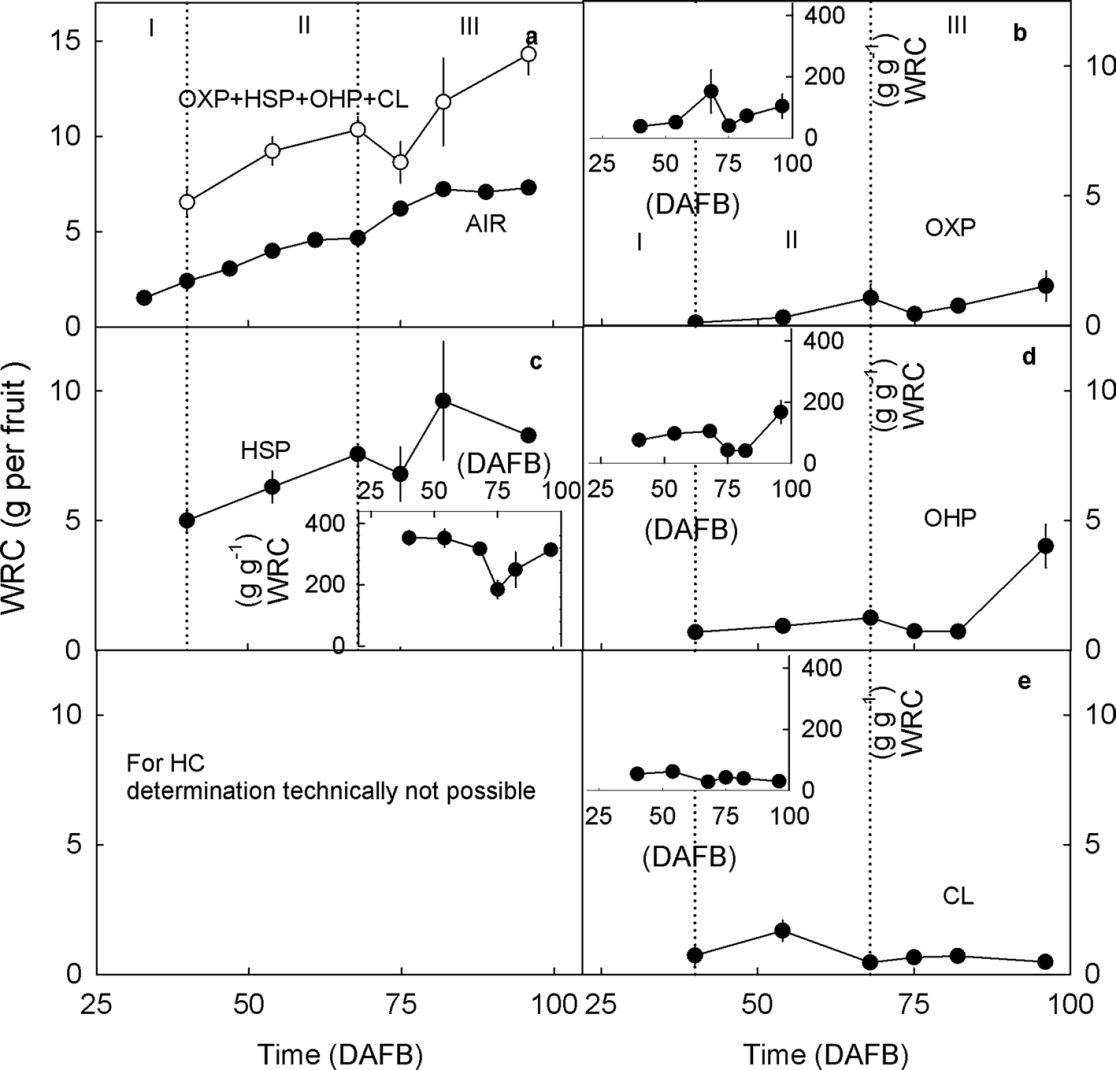
Change in the water retention capacity (WRC) of different cell wall fractions extracted from developing sweet cherry fruit. The WRC was calculated on a whole fruit basis (Main graphs **a**–**f**) and a unit dry mass basis of the respective cell wall fraction (Insets **b**–**f**). **a** Alcohol-insoluble cell wall residue (AIR) and sum of oxalate-soluble pectins (OXP), HCl-soluble pectins (HSP), NaOH-soluble pectins (OHP), and cellulose (CL). **b** OXP. **c** HSP. **d** OHP. **e** HC. **f** CL. Due to the absence of significant swelling, the WRC of the HC cannot be determined reliably. Therefore, the HC data are omitted from this comparison

On a whole-fruit basis, the water retention capacity of the AIR and, even more so, the sum of the intrinsic water retention capacities of the OXP, HSP, OHP and CL fractions all increased during fruit development (Fig. 8a). In this summation, the WSP and the HC were both excluded. The WSP is solubilized in the supernatant. The HC fraction does not swell which makes it impossible to quantify its water retention capacity reliably. On average, the sum of the water retention capacities of the individual fractions was about 2.0-fold greater than that of the AIR. The increase in the water retention capacity was primarily due to an increase in the HSP, followed by an increase in the OHP and the OXP fractions (Fig. 8b, c, d). The water retention capacity of the CL fraction remained about constant and at a low level (Fig. 8e).

### Swelling pressure

**Fig. 9:**
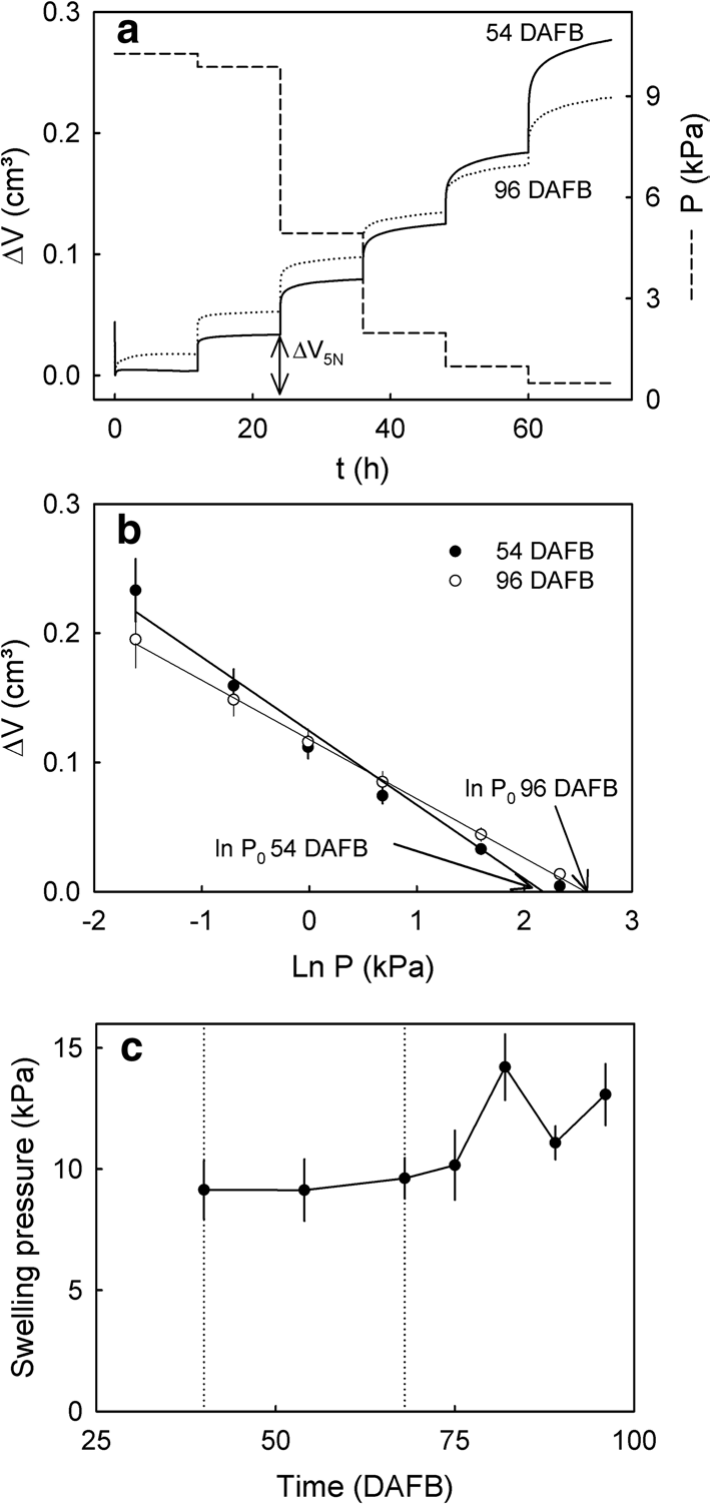
Swelling of cell wall materials extracted from ‘Regina’ sweet cherry 54 and 96 days after full bloom (DAFB) when incubated in deionized water. Swelling was quantified in vitro as the change in volume (∆V) at different pressures (P) using a custom-built pressure chamber. Extracted cell wall material was incubated in water to induce swelling. The ∆V of the swollen cell walls after loading the cell wall with different pressures was quantified. **a** Time course of water-induced swelling of cell walls when the applied pressure was decreased stepwise from 10.3 to 0.1 kPa. At each pressure step, the pressure was held constant for 12 h to allow equilibration of cell wall swelling. **b** Relationship between the swelling of cell walls (∆V) at equilibrium and the applied pressure. The swelling pressure P_0_ corresponds to the pressure at which no swelling occurs. The value P_0_ was estimated as the *x*-axis intercept of a regression line fitted through a plot of ∆V vs. ln P. The regression equation was $$\Delta V = - \,0.37 \, \left( { \pm \, 0.02} \right) \times \, \ln \, P \, \left( {kPa} \right) + 1.42 \, \left( { \pm \, 0.04} \right),\,r^{2} = 0.97^{***}$$ **c** Developmental time course of the change in swelling pressure P_0_. Time scale in days after full bloom (DAFB)

The swelling of the cell wall extracts depended on the applied pressure. Stepwise decreases in the applied pressure allowed the cell wall extracts to swell (Fig. 9a). Swelling was similar for extracts prepared from fruit at 54 and at 96 DAFB. The changes in volume were linearly related to the natural logarithm of the applied pressures (Fig. 9b). The extrapolated X-axis intercepts define the pressures required to prevent any swelling of the cell wall, i.e., the swelling pressure. Swelling pressure was low throughout development and increased towards maturity (Fig. 9c). Furthermore, only small differences were obtained between the swelling pressures of the different cell wall fractions (Table 2). The OHP had the highest swelling pressure, the other fractions did not differ significantly.

**Table 2:** The in vitro swelling pressure of different fractions of extracted cell walls at 96 days after full bloom

Fraction	Swelling pressure (kPa)
OXP	10.5 ± 0.3 a^a^
HSP	7.7 ± 1.2 a
OHP	14.0 ± 0.2 b
HC	10.0 ± 0.8 a
CL	9.41 ± 0.6 a

The sum of the oxalate-soluble pectin (OXP), HCl-soluble pectin (HSP), NaOH-soluble pectin (OHP), hemicellulose (HC) and cellulose (CL) fractions comprised 80% of the alcohol-insoluble residue (AIR) of mature sweet cherry fruit. Data represent means ± SE. The swelling pressure was determined on 20 mg samples of each fraction

^a^Mean separation within columns by Tukey’s Studentized range test, *P* < 0.05

## Discussion

Important findings are:

1. Cell wall deposition does not keep pace with fruit growth.
2. The swelling potential of cell walls increases during development.
3. Across all developmental stages and across all cell wall fractions, the swelling pressures were low, relative to the very negative osmotic potentials of the fruit.

### Cell wall deposition does not keep pace with fruit growth

Cell wall deposition lags behind the increase in fruit fresh mass. This observation was consistent among all cultivars investigated. It is also consistent with earlier reports for ‘Biggarreau Burlat’ (Batisse et al. 1994) and ‘Sweetheart’ and ‘New Star’ sweet cherry (Salato et al. 2013). Apparently, the increase in fruit fresh mass distributed (‘diluted’) the cell wall mass throughout a steadily increasing fruit volume. This result is expected, because the stage III volume growth of the mesocarp is driven primarily by cell expansion (bigger cells) rather than by cell division (more cells) (Tukey and Young 1939; Olmstead et al. 2007). It is interesting that the ‘dilution’ of cell wall material by fruit volume growth did not affect all the cell wall fractions to the same extent. In particular, the HC and CL fractions and within the pectins the HSP, followed by the OHP were more strongly ‘diluted’. There was no ‘dilution’ of the WSP or the OXP. The composition of the major pectin fraction of the ‘Regina’ fruit used in our study was similar to that in earlier reports. In ‘Regina’, the HSP was the largest contributor (31%) to total pectins whereas the OXP fraction was the smallest (18% of total pectins). Barbier and Thibault (1982) reported 38% HSP and 19% OHP for ‘Bigarreaux Napoléon’ using the same extraction method. The percentages of WSP and OXP were about the same in ‘Bigarreaux Napoléon’ as in ‘Regina’ in this study.

On a fresh weight basis, the decrease in HSP and, to a lesser extent, in OHP accounted for the decrease of the AIR. This is consistent with studies by Basanta et al. (2013, 2014) who reported the largest decrease to occur in tightly-bound pectins which are represented by the Na_2_CO_3_-soluble fraction. Similar results were reported by Choi et al. (2002) and Salato et al. (2013). Also, the low percentage of the WSP and its increase towards maturity is consistent with earlier reports (Choi et al. 2002; Basanta et al. 2014). According to Ponce et al. (2010), the increase in the WSP may have been due to a weakening of the crosslinking of pectins by Ca leading to a solubilization of pectins.

Our findings have two important consequences. First, the cell walls are increasingly strained due to the increase in fruit mass particularly during stage III development. During stage III, fruit mass increases primarily due to increase in cell size and, to a lesser extent, in cell number. In contrast, growth in stages I and II is primarily accounted for by increases in cell number. Indeed, cell wall and tissue strain, and hence stress, in the fruit increased markedly during stage III development. The increasing stress is indexed by the gaping of a ‘slit’ wound in a fruit, made with a razor blade (Grimm et al. 2012). The strain and resulting stress in the cell walls are important in the cracking of fleshy fruit. Strain generates the stress, which is the driving force for the propagation of microcracks to form macrocracks (Schumann et al. 2019). However, the cell wall strain did not result in a decrease in cell wall thickness as one might expect. Indeed, cell wall thickness (between healthy, living, turgid cells) increased to 54 DAFB and then remained constant to 96 DAFB. This, despite of a 4.8-fold increase in fruit fresh mass (see Fig. 1a, d). At the same time, the mass of AIR per g fresh mass decreased indicating that the increase in fresh mass resulted in a ‘dilution’ of the cell wall material. The discrepancy between these results may be related to the observation that cell wall thickness represents the thickness of the epidermal (anticlinal) cell walls in the skin (Fig. 1d), whereas the cell wall mass is primarily determined by the cells of the flesh (Fig. 1b, c; Table 1). However, in the flesh, stage III growth (after 68 DAFB) is primarily due to cell expansion. Only during stage I, does cell division take place in the mesocarp (Tukey and Young 1939; Olmstead et al. 2007). In contrast, the fruit skin undergoes continuing cell division into stage III growth (Knoche et al. 2004).

Second, the ‘dilution’ of the cellulose fraction of the cell wall material indicates a likely weakening of the cell wall. The cellulose fraction confers the structural strength and rigidity to the cell wall composite. In contrast, the pectins and hemicelluloses contribute to the cell wall’s plasticity and viscoelasticity (Chanliaud et al. 2002).

### Swelling of cell walls increases during development

The extent of cell wall swelling in sweet cherries was similar to that in other fruitcrop species (Redgwell et al. 1997). For example, for the AIR of pumpkin, De Escalada Plá et al. (2007) reported a swelling capacity of 42 ml g^−1^, a water holding capacity of 43 g g^−1^ and a water retention capacity of 44 g g^−1^. Similarly, for apple the water retention capacity was between 25 and 48 g g^−1^ depending on the extraction procedure (Vetter and Kunzek 2003). Furthermore, marked swelling of extracted cell walls was also reported for plum, persimmon, strawberry (Redgwell et al. 1997), kiwi (Redgwell et al. 1997; Fullerton 2015) and tomato (Shomer et al. 1991; Redgwell et al. 1997; Cantu et al. 2008). Significantly lower swelling capacities and water retention capacities were measured by Figuerola et al. (2005) for concentrates of apple and citrus fiber (no AIR) but their extraction procedures were different. Thus, it is fair to conclude the swelling of sweet cherry cell walls is within the range observed for other fruitcrop species.

That cell wall swelling increases during maturation is typical of fruit that, when ripe, have soft/melting textures. This observation holds for sweet cherry and also for persimmon, avocado, blackberry, strawberry and European plum (Redgwell et al. 1997). Cell wall swelling is the result of the absorption of water into voids within the cell wall. These voids are left behind after solubilization of pectins from the cellulose/hemicellulose network (Redgwell et al. 1997). According to Raghavendra et al. (2004), the water absorption depends on the chemical, physical and microstructural properties of the entire cell wall network. Large values of water holding capacity and water retention capacity are expected for pectins that are readily solubilized in water (Basanta et al. 2013). In particular, hydrophilic polysaccharides such as rhamnogalacturonan I, increase water absorption and swelling (de Escalada Plá 2007). For example, in our study, the swelling capacity and the water retention capacity of the HC and CL were lower than of the pectins. An occlusion of pectins by HC or CL would decrease swelling. This is not unlikely. Recent evidence suggests that the CL and HC fractions may contain some pectins (Broxterman and Schols 2018). For fruit, the pectin content in the CL ranges from 5% of the total content of galacturonic acid of the cell wall in strawberry, to 10% in tomato (Broxterman and Schols 2018). If this was also the case in sweet cherry in our study, these hypothetical pectins in the HC and CL fractions did not contribute to swelling as indexed by the low intrinsic swelling capacity and water retention capacity of the HC and CL. Thus, our conclusion that the swelling was mostly due to the HSP and OHP (and possibly to the WSP) remains unaffected.

Although the in vitro swelling capacity and water retention capacity of the AIR were significantly correlated with the in vivo cell wall swelling, we observed a discrepancy between in vivo and in vitro assessments of swelling of the AIR, particularly during stage III. The microscopic in vivo assessments indicated continuing swelling, whereas the in vitro assays revealed little further change. This discrepancy may be accounted for by the sharp increase in the WSP fraction. This more than doubled from the stage II/III transition to late stage III. This fraction will be in solution and, hence, in the supernatant in the in vitro assays. Therefore, it will not contribute to the in vitro swelling as assessed by determination of the swelling capacity, the water holding capacity and the water retention capacity.

That the sum of the swelling capacities and the water retention capacities of the individual cell wall fractions exceeded that of the AIR is not surprising. The swelling capacities and the water retention capacities of the individual cell wall fractions characterize the ‘intrinsic’ swelling behavior in vitro of the extracted fraction in the absence of interactions with other cell wall constituents that occur in the cell wall composite in vivo. Consequently, the absence of swelling in vitro indicates that there will also be no swelling of the respective fraction in vivo, i.e., when still part of the cell wall composite. However, significant swelling of the extracted fraction in vitro indicates that the respective fraction may contribute to swelling in vivo, i.e., the swelling observed by microscopy of the epidermal cell walls. Whether it indeed contributes to swelling in vivo, will depend on its interaction with other cell wall constituents. For the swelling in vivo, the spatial arrangement of cell wall constituents within the cell wall composite and the interaction with cross-linking ions such as calcium is a critical factor (Basanta et al. 2013).

### Cell wall swelling pressure is very low

The swelling pressure determined in vitro using extracted cell wall material was very low across all developmental stage and also across the different cell wall fractions. This finding is consistent with earlier ones indicating that the swelling of cell walls is a physical process, normally counterbalanced by cell turgor (Grimm and Knoche 2015; Schumann and Knoche 2020). Swelling occurs when turgor is lost, regardless of whether this is the result of turgor release following imposition of a freeze/thaw cycle or by plasmolyzing epidermal cells by exposure to a hypertonic osmoticum (Schumann and Knoche 2020). Because swelling pressures are very low, even the low cell turgors in stage III sweet cherry fruit are quite sufficient to prevent the swelling of cell walls in vivo (Schumann et al. 2014). Hence, cell wall swelling pressure is not a significant component of the water potential of sweet cherry fruit. That the sum of the swelling pressures of the individual extracted cell wall fractions exceeded that of the AIR is accounted for by the lack of interaction of the extracted fraction with other cell wall constituents as explained above for the swelling capacity and the water retention capacity.

## Conclusion

Three explanations can be offered that contribute to the increase in cracking susceptibility of developing sweet cherry fruit reported in the literature (Christensen 1973). First, the low rate of cell wall deposition during growth results in a ‘dilution’ of cell walls as ongoing expansion growth increases cell wall strain. This results in a buildup of cell wall stress, which represents the driving force for cracking of sweet cherry and other fleshy fruitcrops. Second, the compositional changes that occur during cell wall development render the fruit flesh and skin less rigid and structurally weaker due to a relative decrease in the cellulose fraction. In addition, a general increase in the pectins fraction renders the cell walls more plastic and viscoelastic. Third, cell wall swelling increases due, in particular, to relative increases in the pectin fraction. As a result, cell:cell adhesion decreases making cells more susceptible to schizogony the separation of adjacent cells along the line of the middle lamella (Brüggenwirth and Knoche 2017; Schumann et al. 2019). This is the dominant fracture mode for rain cracking in sweet cherry.

### *Author contribution statement*

CS and MK conceived and designed the experiments. CS, SS and LE conducted the measurements. CS analyzed the data. CS and MK wrote the manuscript. All authors read and approved the manuscript.

## Abbreviations

AIR: Alcohol-insoluble residue
ES: Epidermal skin section(s)
WSP: Water-soluble pectins
OXP: Oxalate-soluble pectins
HSP: HCl-soluble pectins
OHP: NaOH-soluble pectins
CL: Cellulose fraction
HC: Hemicellulose fraction
TP: Total pectins

## References

[CR1] Alkio M, Jonas U, Sprink T, van Nocker S, Knoche M (2012). Identification of putative candidate genes involved in cuticle formation in *Prunus avium* (sweet cherry) fruit. Ann Bot.

[CR2] Barbier M, Thibault JF (1982). Pectic substances of cherry fruits. Phytochemistry.

[CR3] Basanta MF, de Escalada PM, Stortz CA, Rojas AM (2013). Chemical and functional properties of cell wall polymers from two cherry varieties at two developmental stages. Carbohydr Polym.

[CR4] Basanta MF, Ponce NMA, Salum ML, Raffo MD, Vicente AR, Erra-Balsells R, Stortz CA (2014). Compositional changes in cell wall polysaccharides from five sweet cherry (*Prunus avium* L.) cultivars during on-tree ripening. J Agric Food Chem.

[CR5] Batisse C, Fils-Lycaon B, Buret M (1994). Pectin changes in ripening cherry fruit. J Food Sci.

[CR6] Batisse C, Buret M, Coulomb PJ (1996). Biochemical Differences in cell wall of cherry fruit between soft and crisp fruit. J Agric Food Chem.

[CR7] Borve J, Sekse L, Stensvand A (2000). Cuticular fractures promote postharvest fruit rot in sweet cherries. Plant Dis.

[CR8] Broxterman SE, Schols HA (2018). Interactions between pectin and cellulose in primary plant cell walls. Carbohydr Polym.

[CR9] Brüggenwirth M, Knoche M (2017). Cell wall swelling, fracture mode, and the mechanical properties of cherry fruit skins are closely related. Planta.

[CR10] Brüggenwirth M, Fricke H, Knoche M (2014). Biaxial tensile tests identify epidermis and hypodermis as the main structural elements of sweet cherry skin. Ann Bot Plants.

[CR11] Cantu D, Vicente AR, Greve LC, Dewey FM, Bennett AB, Labawitch JM, Powell ALT (2008). The intersection between cell wall disassembly, ripening, and fruit susceptibility to *Botrytis cinerea*. Proc Natl Acad Sci USA.

[CR12] Chanliaud E, Burrows KM, Jeronomidis G, Gidley MJ (2002). Mechanical properties of primary plant cell wall analogues. Planta.

[CR13] Choi C, Toivonen P, Wiersma PA, Kappel F (2002). Differences in levels of pectic substances and firmness in fruit from six sweet cherry genotypes. J Am Pomol Soc.

[CR14] Christensen JV (1973). Cracking in cherries. VI. Cracking susceptibility in relation to the growth rhythm of the fruit. Acta Agric Scand.

[CR15] de Escalada Pla MF, Ponce NMA, Stortz CA, Rojas AM, Gerschenson LN (2007). Composition and functional properties of enriched fibre products obtained from pumpkin (*Cucurbita moschata*, Duchesne ex Poiret). LWT-Food Sci Technol.

[CR16] Figuerola F, Hurtado ML, Estévez AM, Chiffelle I, Asenjo F (2005). Fibre concentrates from apple pomace and citrus peel as potential fibre sources for food enrichment. Food Chem.

[CR17] Fügel R, Carle R, Schieber A (2004). A novel approach to quality and authenticity control of fruit products using fractionation and characterisation of cell wall polysaccharides. Food Chem.

[CR18] Fügel R, Schieber A, Carle R (2006). Determination of the fruit content of cherry fruit preparations by gravimetric quantification of hemicellulose. Food Chem.

[CR19] Fullerton CG (2015) Kiwifruit softening: a cell wall study. Dissertation, University of Auckland.

[CR20] Glenn GM, Poovaiah BW (1989). Cuticular properties and postharvest calcium applications influence cracking of sweet cherries. J Am Soc Hortic Sci.

[CR21] Grimm E, Knoche M (2015). Sweet cherry skin has a less negative osmotic potential than the flesh. J Am Soc Hortic Sci.

[CR22] Grimm E, Peschel S, Becker T, Knoche M (2012). Stress and strain in the sweet cherry fruit skin. J Am Soc Hortic Sci.

[CR23] Grimm E, Hahn J, Pflugfelder D, Schmidt M, van Dusschoten D, Knoche M (2019). Localized bursting of mesocarp cells triggers catastrophic fruit cracking. Hortic Res.

[CR24] Grimm E, Pflugfelder D, Hahn J, Schmidt MJ, Dieckmann H, Knoche M (2020). Spatial heterogeneity of flesh-cell osmotic potential in sweet cherry affects partitioning of absorbed water. Hortic Res.

[CR25] Herrmann K (2001). Inhaltsstoffe von Obst und Gemüse.

[CR26] Khanal BP, Grimm E, Knoche M (2011). Fruit growth, cuticle deposition, water uptake, and fruit cracking in jostaberry, gooseberry, and black currant. Sci Hortic.

[CR27] Knoche M, Lang A (2017). Ongoing growth challenges fruit-skin integrity. Crit Rev Plant Sci.

[CR28] Knoche M, Peschel S (2006). Water on the surface aggravates microscopic cracking of the sweet cherry fruit cuticle. J Am Soc Hortic Sci.

[CR29] Knoche M, Beyer M, Peschel S, Oparlakov B, Bukovac MJ (2004). Changes in strain and deposition of cuticle in developing sweet cherry fruit. Physiol Plant.

[CR30] Knoche M, Khanal BP, Stopar M (2011). Russeting and microcracking of 'Golden Delicious' apple fruit concomitantly decline due to Gibberellin A4+7 application. J Am Soc Hortic Sci.

[CR31] Kondo S, Danjo C (2001). Cell wall polysaccharide metabolism during fruit development in sweet cherry 'Satohnishiki' as affected by gibberellic acid. J Japan Soc Hortic Sci.

[CR32] Lichter A, Dvir O, Fallik E, Cohen S, Golan R, Shemer Z, Sagi M (2002). Cracking of cherry tomatoes in solution. Postharvest Biol Technol.

[CR33] Maguire K, Lang A, Banks NH, Hall A, Hopcroft D, Bennett R (1999). Relationship between water vapour permeance of apples and micro-cracking of the cuticle. Postharvest Biol Technol.

[CR34] Mrozek RF, Burkhardt TH (1973). Factors causing prune side cracking. Trans Am Soc Agric Eng.

[CR35] Olmstead JW, Iezzoni AF, Whiting MD (2007). Genotypic differences in sweet cherry fruit size are primarily a function of cell number. J Am Soc Hortic Sci.

[CR36] Opara LU, Studman CJ, Banks NH (1997). Fruit skin splitting and cracking. Hortic Rev.

[CR37] Peschel S, Knoche M (2005). Characterization of microcracks in the cuticle of developing sweet cherry fruit. J Am Soc Hortic Sci.

[CR38] Ponce NMA, Ziegler VH, Stortz CA, Sozzi GO (2010). Compositional changes in cell wall polysaccharides from Japanese plum (*Prunus salicina* Lindl.) during growth and on-tree ripening. J Agric Food Chem.

[CR39] Raghavendra SN, Rastogi NK, Raghavarao KSMS, Tharanathan RN (2004). Dietary fiber from coconut residue: effects of different treatments and particle size on the hydration properties. Eur Food Res Technol.

[CR40] Redgwell RJ, MaxRae E, Hallett I, Fischer M, Perry J, Harker R (1997). In vivo and in vitro swelling of cell walls during fruit ripening. Planta.

[CR41] Rombouts FM, Thibault JF (1986). Feruloylated pectic substances from sugar-beet pulp. Carbohydr Res.

[CR42] Salato GS, Ponce NMA, Raffo MD, Vicente AR, Stortz CA (2013). Developmental changes in cell wall polysaccharides from sweet cherry (*Prunus avium* L.) cultivars with contrasting firmness. Postharvest Biol Technol.

[CR43] Saulnier L, Thibault JF (1987). Extraction and characterization of pectic substances from pulp of grape berries. Carbohydr Polym.

[CR44] Schumann C, Knoche M (2020). Swelling of cell walls in mature sweet cherry fruit: Factors and mechanisms. Planta.

[CR45] Schumann C, Schlegel HJ, Grimm E, Knoche M, Lang A (2014). Water potential and its components in developing sweet cherry. J Am Soc Hort Sci.

[CR46] Schumann C, Winkler A, Brüggenwirth M, Köpcke K, Knoche M (2019). Crack initiation and propagation in sweet cherry skin: a simple chain reaction causes the crack to ‘run’. PLoS ONE.

[CR47] Shomer I, Frenkel H, Polinger C (1991). The existence of a diffuse electric double layer at cellulose fibril surfaces and its role in the swelling mechanism of parenchyma plant cell walls. Carbohydr Polym.

[CR48] Sozzi GO, Greve LC, Prody GA, Labavitch JM (2002). Gibberellic acid, synthetic auxins, and ethylene differentially modulate α-L-arabinofuranosidase activities in antisense 1-aminocyclopropane-1-carboxylic acid synthase tomato pericarp discs. Plant Physiol.

[CR49] Tukey HB, Young JO (1939). Histological study of the developing fruit of the sour cherry. Bot Gaz.

[CR50] Vetter S, Kunzek H (2003). The influence of suspension solution conditions on the rehydration of apple cell wall material. Eur Food Res Technol.

[CR51] Winkler A, Ossenbrink M, Knoche M (2015). Malic acid promotes cracking of sweet cherry fruit. J Am Soc Hortic Sci.

[CR52] Winkler A, Peschel S, Kohrs K, Knoche M (2016). Rain cracking in sweet cherries is not due to excess water uptake but to localized skin phenomena. J Am Soc Hortic Sci.

[CR53] Yapo BM, Koffi KL (2008). The polysaccharide composition of yellow passion fruit rind cell wall: chemical and macromolecular features of extracted pectins and hemicellulosic polysaccharides. J Sci Food Agric.

[CR54] Yapo BM, Lerouge P, Thibault JF, Ralet MC (2007). Pectins from citrus peel cell walls contain homogalacturonans homogenous with respect to molar mass, rhamnogalacturonan I and rhamnogalacturonan II. Carbohydr Polym.

